# Randomized controlled trials of malaria intervention trials in Africa, 1948 to 2007: a descriptive analysis

**DOI:** 10.1186/1475-2875-10-61

**Published:** 2011-03-15

**Authors:** Vittoria Lutje, Annette Gerritsen, Nandi Siegfried

**Affiliations:** 1 International Health Research Group, Liverpool School of Tropical Medicine, Liverpool, UK; 2 Department of Public Health, University of Venda, Thohoyandou, South Africa; 3 South African Cochrane Centre, Medical Research Council, Cape Town, South Africa

## Abstract

**Background:**

Nine out of ten deaths from malaria occur in sub-Saharan Africa. Various control measures have achieved some progress in the control of the disease, but malaria is still a major public health problem in Africa. Randomized controlled trials (RCTs) are universally considered the best study type to rigorously assess whether an intervention is effective. The study reported here provides a descriptive analysis of RCTs reporting interventions for the prevention and treatment of malaria conducted in Africa, with the aim of providing detailed information on their main clinical and methodological characteristics, that could be used by researchers and policy makers to help plan future research.

**Methods:**

Systematic searches for malaria RCTs were conducted using electronic databases (Medline, Embase, the Cochrane Library), and an African geographic search filter to identify RCTs conducted in Africa was applied. Results were exported to the statistical package STATA 8 to obtain a random sample from the overall data set. Final analysis of trial characteristics was done in a double blinded fashion by two authors using a standardized data extraction form.

**Results:**

A random sample of 92 confirmed RCTs (from a total of 943 reports obtained between 1948 and 2007) was prepared. Most trials investigated drug treatment in children with uncomplicated malaria. Few trials reported on treatment of severe malaria or on interventions in pregnant women. Most trials were of medium size (100-500 participants), individually randomized and based in a single centre. Reporting of trial quality was variable. Although three-quarter of trials provided information on participants' informed consent and ethics approval, more details are needed.

**Conclusions:**

The majority of malaria RCT conducted in Africa report on drug treatment and prevention in children; there is need for more research done in pregnant women. Sources of funding, informed consent and trial quality were often poorly reported. Overall, clearer reporting of trials is needed.

## Background

Almost 90% of all malaria cases occur in sub-Saharan Africa, with the major burden on children under five years of age and pregnant women [1]. Current measures to control malaria show some degree of success: more than one-third of malaria-endemic countries, including nine African countries, have reported a reduction of malaria cases of >50% between 2000 and 2008 [2,3]. However, malaria remains a major public health problem in Africa, and more work is needed to evaluate new interventions and those currently in use.

Randomized controlled trials (RCTs) provide unbiased estimates of the effects of an intervention [4], and allow formal synthesis of results between trials in systematic reviews and meta-analysis. Up-to-date information on completed, ongoing, and planned RCTs for malaria interventions is needed to inform policy and to plan future research, but an overview of these RCTs is not currently available. Although several studies [5-7] have reported on different methodological aspects of RCTs conducted in Africa, and on their relevance to the burden of disease of the local populations, a comprehensive analysis of clinical and methodological characteristics of malaria RCTs run in Africa has been lacking. This comprehensive evaluation can help determine which areas of malaria research have been predominant or overlooked, and assess whether RCTs have effectively covered the health needs of the whole population. In addition, an examination of the methodological characteristics and quality of RCTs can be used to highlight training needs for trialists and other issues related to ethical approval and participants' consent. For this analysis a database of RCTs of malaria prevention and treatment run in Africa was prepared and the clinical characteristics and methodology of these trials were assessed and reported in this article.

This work was undertaken under the umbrella of the Pan African Clinical Trials Registry [8], which was established with support from the European and Developing Countries Clinical Trials Partnership.

## Methods

On 31^st ^August 2007 a database of malaria trials prepared for the Cochrane Infectious Diseases Group (CIDG) was searched to identify RCTs conducted in Africa. The CIDG malaria trials database is populated from searches in Medline, Embase, Cochrane CENTRAL and LILACS, to identify RCTs. The search strategy included the terms "malaria", "plasmodium", and the Cochrane sensitive filter to identify RCTs [9]. Trials conducted in African countries were identified by applying an African geographic search filter [10]. These records were exported into an MS Excel spreadsheet and categorized using the following variables: malaria prevention or treatment, type of intervention (drugs, bed nets, nutritional supplements, vaccines), type of participants (adults, children, pregnant women) and type of malaria (asymptomatic, uncomplicated or severe). Records were then exported to the statistical package STATA 8 to obtain a random sample from the overall data set. The random sample was meant to be 10% and intentionally over-sampled to ensure that there would be sufficient RCTs for data extraction. To ensure the random sample was broadly representative of the initial dataset, variables from abstracts of the records included in the two sets were extracted and compared their frequencies. Two authors (VL and AG) then screened the abstracts of the articles included in the random sample in a blinded fashion to identify RCTs. A third author (NS) acted as an arbiter in case of disagreement. Full articles for the confirmed RCTs were retrieved. A data extraction form was developed and pilot-tested on ten articles. VL and AG extracted data using this standardized data extraction form and a third author (NS) resolved disagreements when these arose. The full set of variables extracted from these trials is shown in Additional file 1. One author (VL) examined the data extraction forms and entered data in MS Excel for final analysis. Results are presented as frequency of variables, calculated in MS Excel.

## Results

### Search results

**Figure 1:**
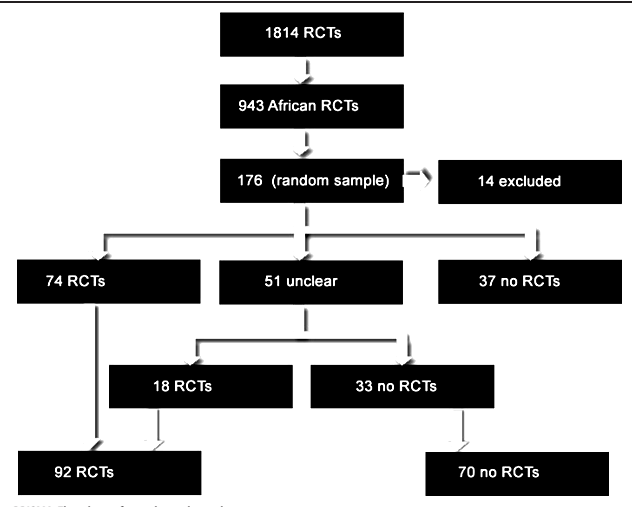
**PRISMA Flowchart of search results and assessment**.

A total of 1,814 records describing malaria trials between 1948 and August 2007 was identified; 943 articles (51.9%) were reports of trials in Africa (Figure 1). A random sample of 176 records was drawn from the total dataset. After reading each available abstract from the random sample, and excluding 14 reports (non-RCTs or not taking place in Africa), seventy-four studies described as RCTs were considered eligible for inclusion in the analysis A further 37 records were not eligible as they were not RCTs, and for a further 51 records it was not possible to clearly determine eligibility from the abstract. Two authors (VL and AG) read the full text of these 51 "unclear" records and a further 18 RCTs were identified for inclusion. The final number of RCTs included in the analysis was 92 (52.2% of the random sample of 176); these RCTs were run between 1977 and 2007. The full text of these 92 trials was retrieved for further analysis.

### Location of trials, principal investigators and funding

**Figure 2:**
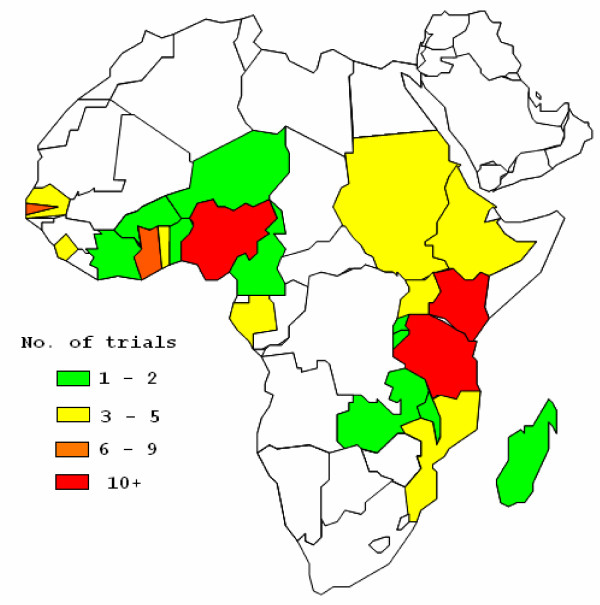
**Geographical distribution of malaria RCT run in Africa**.

Figure 2 shows the location of the trials included in the final analysis. RCTs took place in a total of 27 African countries. Countries hosting more than five trials were Kenya (13), Nigeria (10), Tanzania (10), the Gambia (8), and Ghana (6). Other African countries hosted between one and four RCTs. Over 60% of RCTs (n = 58) were conducted in rural areas, 27 were run in town and cities, one trial had both urban and rural sites, and for 5 of them the location was unclear.

The principal investigator (PI) was not clearly declared in every article. When the address of the PI was not reported the location of the corresponding author was used. Thirty-seven PIs lived in African countries: Nigeria (8), Sudan (4), Kenya (4), Tanzania (3), Burkina Faso (2), Ethiopia (2), Ghana (2), Mozambique (2), the Gambia (2), Uganda (2), Benin (1), Cameroon (1), Guinea Bissau (1), Madagascar (1), Niger (1), and Togo (1). Forty-two PIs lived in non-African countries with most PIs based in the USA (10) and the UK (9), Denmark (5) and Switzerland (5). It was not possible to determine the country of residence of the PI in 13 reports. Source of funding was not reported in one fifth of the trials (n = 19) and even when reported, it was not always possible to determine exactly the nature of the funding agency and the relative contributions of governments, non-government organizations and pharmaceutical companies.

### Characteristics of included trials

Fifty-three of the 92 included trials evaluated a treatment intervention for malaria and 38 trials reported on malaria preventive measures; one trial reported both prevention and treatment.

For the 53 trials of malaria treatment, 44 reported on treatment of acute uncomplicated malaria, seven reported on severe malaria, one on asymptomatic malaria and one report was unclear. All but one of the trials reported on a drug intervention.

Of the 38 trials of malaria prevention, 10 reported on drug interventions and 10 on the use of bed nets or other physical barriers, six reported on nutritional supplements, six on vaccines, five on combinations of nutritional supplements and drugs and one trial reported on a combination of bed nets and drug prophylaxis.

**Figure 3:**
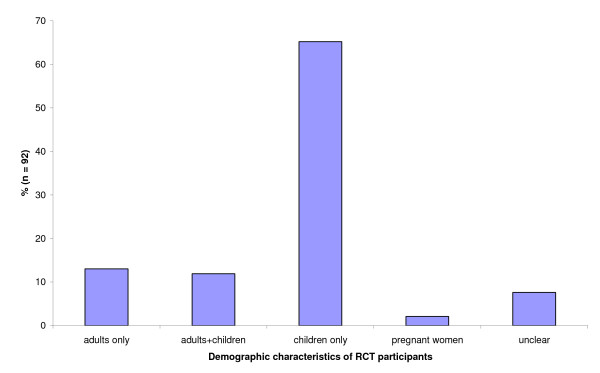
**Demographic characteristics of trials' participants**.

The demographic characteristics of the participants in the 92 trials are shown in Figure 3.

### Sample size and methodological quality

Ninety trials were parallel randomized and two had a crossover design. Seventy-two trials were randomized at the individual level, 14 trials were cluster randomized (mostly describing large scale interventions, such as use of bed nets or nutritional supplements) and for four trials the level of randomization was unclear. Two-thirds (62 of the total) compared two interventions, 16 trials compared three interventions, and 14 compared four or more interventions.

Assessment of the methodological quality of RCTs was based on four items: allocation generation, allocation concealment, blinding and loss to follow up. RCTs from the random sample published between 1997 and 2007 were considered for this analysis (n = 60). Results are shown in Table 1. Allocation generation was considered adequate (as done by using a random numbers table or electronically generated) in 35 RCTs, but methods were not reported in 21 RCTs. Many RCTs (36) did not mention methods of allocation concealment or of blinding of participants or intervention providers (27). Loss to follow up was accounted for in most RCTs (49).

**Table 1:** Assessment of trial quality for African malaria RCTs published between 1997 and 2007. N = 60 trials

Items	Adequate	Not adequate	Unclear	Not done
Allocation generation	35	3	21	1
Allocation concealment	14		36	10
Blinding	23		27	10
Loss to follow up	49	n/a	11	

**Figure 4:**
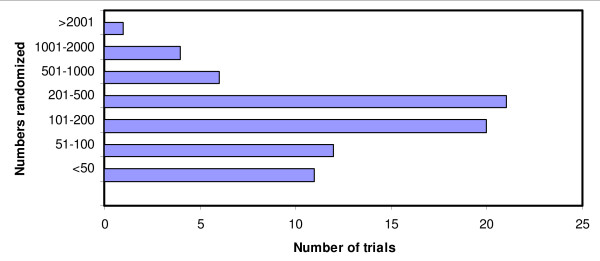
**Trials size - Number of participants randomized**.

The number of participants included in the 72 individually randomized malaria trials is shown in Figure 4. The lowest number of participants was 12 (two RCTs both reporting on malaria drug treatment in adults), and the highest number of participants was 22,000 (a study of nutritional supplements in malaria prevention in children in Ghana) with a median of 210 participants per trial.

### Ethics and informed consent

Of the 92 included RCTs, 35 had been approved by a national ethics committee, 31 had received the approval of both a national and an international ethics committee, and 26 did not report any detail about ethical approval. Older RCTs were more likely not to report on ethical approval: of the 33 studies run between 1977 and 1997 (the first 20 years of our random sample), over half (17) did not mention this characteristic, in contrast to only nine out of 59 that were run between 1998 and 2007.

Thirty-nine RCTs reported that participants' informed consent had been obtained but did not specify any details, 21 reported obtaining written consent, five reported oral consent and in three cases both oral and written consent was obtained. Participants' informed consent was not reported in 25 RCTs.

## Discussion

This study was designed to obtain information on the clinical and methodological characteristics of RCTs conducted in Africa on malaria interventions. This information is needed to plan and implement future malaria clinical trials, to suggest research areas that may have been overlooked, and to point to methodological aspects that need to be addressed when planning future activities.

This was a novel analysis and no similar comprehensive study of African malaria RCTs has been done before.

More than half of the total malaria trials identified in the initial search were conducted in Africa, a finding consistent with the high local burden of disease but also indicative of high levels of research activity, as shown for other infectious and parasitic diseases affecting Africa [5]. More RCTs were run in large countries such as Nigeria, Tanzania or Kenya, but the size of a country or its population were not directly indicative of numbers: smaller countries (Ghana, the Gambia) also contributed many RCTs, possibly as a result of a better health infrastructure or the presence of well established research institutions (such as the UK Medical Research Council Laboratories based in the Gambia). A Wellcome Trust report [11] similarly reported a high number of malaria research studies and publications between 1995 and 1997 originating from Kenya, Tanzania, Nigeria and the Gambia.

Approximately half of PIs in the studies analysed were based in African countries (mainly Nigeria, Kenya, Sudan and Tanzania); it is possible that some PIs were of African origin but resident and working outside the continent, as it has been reported for HIV/AIDS researchers [12] Most authors based outside Africa were resident in the USA and UK, but several had an address in Switzerland or in Denmark, smaller countries with a long-standing tradition of international collaborations. The presence of locally based researchers has been found to positively affect the local development of skills and training [7,12] and is in general correlated to research relevant to the particular country.

Information about trial funding and sponsorship was not included, or was poorly reported, in many published trials, and for this reason it has not been included in the final analysis. It has previously been shown that almost all African HIV/AIDS trials are led and funded by international organizations [10] and if the same is true of malaria trials, this can explain why many authors of trial reports are based outside Africa. The nature of funding agencies is also likely to influence priorities for trials and research receiving funding from private industry, for instance, was found to be associated with reduced emphasis on diseases relevant to Africa [7].

### Clinical characteristics

The majority of RCTs reported on drug treatment of children suffering from uncomplicated malaria, in agreement with the high burden of malaria in the under-fives [13] and with the development and testing of novel drug regimens such as artemisinin-based combination therapy (ACT). ACT was included in World Health Organization malaria guidelines in 2006 [14,15], prompting many African countries to run clinical trials in order to assess the best drug combination to be included in national policies.

Few RCTs investigated treatment of severe malaria or interventions in pregnant women. The scarcity of clinical trials for severe malaria was noted in a previous study [16], showing that fewer than 10,000 patients have been included in RCTs despite approximately ten million cases of severe malaria occurring annually. Clinical studies in severe malaria are considered difficult to plan and execute [17], and are often characterized by slow enrolment, requiring many sites and several years for completion. Also, after the successful introduction of ACT regimens, patients with severe malaria may simply not be found any more in some countries, as reported in Vietnam [18].

The paucity of RCTs assessing interventions to prevent or treat malaria in pregnancy is a cause for concern, as pregnant women are especially vulnerable to *Plasmodium *and the infection can also affect the unborn child. The scarcity of human studies on the pharmacokinetics, safety and efficacy of anti-malarials in pregnancy has been described previously [19,20], and the need for more well-designed and clearly reported RCTs in pregnant women was highlighted in a recent Cochrane review [21]. Pregnant women are routinely excluded from clinical trials involving the general population to avoid harm to them or to their unborn baby, but when coupled with few clinical trials with only pregnant women as participants, this limits the amount of information available on drug safety and effectiveness. It must be noted that recent research initiatives, such as the Malaria in Pregnancy (MiP) Consortium [22], aim to address this lack of knowledge, and several trials to evaluate interventions for the prevention and treatment of malaria in pregnancy, sponsored by this or other organizations, are currently ongoing.

### Trial methodology

The methodological quality of malaria RCTs published in the decade 1997-2007 was assessed. These RCTs were likely to have been conducted after the publication in 1996 of the original CONSORT guidelines for reporting RCTs [23]. The quality of reporting was not consistent among its different domains: loss to follow-up was accounted for in the majority of RCTs, but allocation concealment was mostly unclear, and generation of allocation sequence and blinding were also not clearly reported or altogether not mentioned in a large proportion of malaria RCTs. Similar poor reporting has been widely reported for RCTs in different medical areas [24,25], not only in those conducted in the first few years after the publication of the CONSORT guidelines but also for recent ones [26,27]. Suboptimal trial reporting is a worldwide concern and indeed a recent analysis of HIV/AIDS trials [12] found that African trials had better reporting for allocation concealment and for random generation than North American trials. Prospective trail registration accompanied by education of trialists about the CONSORT (through instructions provided by trial registers at the time of initial registration) can help.

### Ethics, informed consent

Approximately three-quarters of RCTs reported on approval by an ethics committee and a similar percentage included information on participants' consent to the procedure. Reporting of approval by an ethics committee markedly improved over time and the vast majority of RCTs run in the last decade had received local or international ethics approval. This is an encouraging result overall, and several trial reports also provided a detailed description of the consent procedure; however, 38 reports stated consent had been obtained, but did not provide any details. Similarly, in a recent systematic review of registered trials of malaria, tuberculosis and HIV/AIDS [28], informed consent was reported in 58% of trials. When information is not clearly reported or lacking altogether, the possibility arises that informed consent was not always "truly informed" or "truly voluntary", perhaps because of low literacy levels and other socio-economic and cultural factors, as suggested in a recent analysis of trials conducted in sub-Saharan Africa [29]. Studies from Malawi [30,31] reported that participation into research studies was linked to the hope of benefiting from the material and monetary incentives available, including the ancillary care provided by clinical trials. Incomplete reporting or consent which is not truly informed, (especially where resources are limited) is a problem that needs to be urgently addressed, possibly by strengthening national guidelines on clinical research including ethics review.

### Trial reporting and the need for prospective registration

A recurrent finding in the present analysis was that in many trials, several characteristics were suboptimally reported. Details about funding, PI location, and informed consent were often missing or, if mentioned, were difficult to interpret. Similarly, reporting on the methodology used in many trials was poor. This finding is not unique for malaria RCTs or for trials conducted in Africa, but the problem of difficult interpretation and poor compliance with the CONSORT statement [23,32] needs to be addressed to allow for sound conclusions and evidence-based decision making. There is need for education of trialists about the CONSORT statement as well as stringent requirements for publication in medical and scientific journals.

Strengths of this study include a comprehensive search of the literature using multiple databases and randomly sampling from the total dataset, to ensure a high degree of representativeness. In addition, both the eligibility process and data extraction were conducted by two independent investigators and a third provided quality evaluation. Despite this, findings are limited by the fact that only access to reported data was possible. It is likely that additional trials have been conducted which have not been presented at a conference or reported in the literature [33]. Prospective trial registration ensures that essential information is publicly available before a trial begins, through a set of pre-agreed standards. It also provides a unique trial registration number that makes it easy to identify a specific trial and minimize duplication. Within Africa, the Pan African Clinical Trials Registry [8] is a new initiative that aims to prospectively register all clinical trials run in Africa [34]. Prospective registration of African RCTs, by ensuring that trials authors comply with a series of methodological items, will help improve trial quality, reduce publication bias and facilitate regular updating of analyses similar to the one presented here. Providing regulatory bodies in African countries take steps to encourage trialists to register on the Pan African Clinical Trials Registry, unnecessary duplication of research may be avoided, and better harmonization achieved between ethics, regulatory and registration bodies.

## Conclusions

Most malaria RCTs run in Africa investigated treatment, predominantly with drugs, in children with uncomplicated malaria. Few trials reported on treatment of severe malaria or on interventions in pregnant women, but new initiatives and several new trials are addressing interventions in pregnant women. Most trials were of medium size (100-500 participants), individually randomized and based in a single centre. Reporting of methodological quality of trials was often unclear or inconsistent among its components. Although three-quarter of trials provided information on participants' informed consent and ethics approval, more details and clear reporting are needed. Prospective trial registration will help. These findings are valuable because they provide baseline knowledge of malaria trials that should facilitate the planning and implementation of future trials of malaria interventions in Africa.

## Competing interests

Vittoria Lutje and Nandi Siegfried are members of the Pan African Clinical Trials Registry Working Group.

## Authors' contributions

VL and NS contributed the original idea for the study. VL run the literature searches and designed the data extraction form. VL, AG and NS performed data analyses and interpreted the data. NS prepared the random sample and acted as an arbiter on data extraction. VL drafted the manuscript and all authors revised it critically. All authors read and approved the final manuscript.

## Supplementary Material

Additional file 1 **Data extraction variables for each trial included in the analysis**. A table listing details of the trial characteristics that were extracted from each report included in the article. Click here for file
