# Supplementary material for: Randomized controlled trials of malaria intervention trials in Africa, 1948 to 2007: a descriptive analysis
Source: Malar J. 2011 Mar 15;10:61. doi: 10.1186/1475-2875-10-61 (PMC3064642; doi:10.1186/1475-2875-10-61)
Supplement: Additional file 1 — Data extraction variables for each trial included in the analysis. A table listing details of the trial characteristics that were extracted from each report included in the article. [file 1475-2875-10-61-S1.DOC]

# Additional file 1

## Data extraction variables for each trial included in the analysis

| **Variable** | **Details recorded** |  |
| --- | --- | --- |
| **Clinical malaria** | Uncomplicated  Severe  Asymptomatic |  |
| **Type of participants** | Children only  Adults only  Adults + children  Pregnant women |  |
| **Intervention** | Prevention | Types of interventions :  Drugs (Chemoprophylaxis)  Physical barriers (bednets, clothing)  Vaccines  Nutritional supplements  Drugs + physical barriers  Drugs + nutritional supplements |
|  | Treatment | Types of interventions :  Drugs |
| **Dates, country** | Dates of recruitment of trial participants, study duration, end of follow-up.  Country where the trials was conducted. |  |
| **Location of trial** | Urban  Rural |  |
| **Principal investigator** | Name  Country of residence |  |
| **Number of participants** |  |  |
| **Number of comparisons** |  |  |
| **Risk of bias** | Allocation generation  Allocation concealment  Blinding  Loss to follow-up |  |
| **Randomization** | Individual  cluster |  |
| **Setting** | Individual center  Multicenter |  |
| **Ethical approval** | Local  International  Local + international |  |
| **Consent** | Oral  Written  Oral + written  Yes but method not specified  Not reported |  |
| **Source of funding** | Government  NGO  Government + NGO  Pharma  Inter-agencies |  |
